# Possible Therapies for Hepatocellular Carcinoma—Preparing for the Modern War with the Insidious Enemy

**DOI:** 10.3390/ijms241612536

**Published:** 2023-08-08

**Authors:** Michał P. Wasilewicz

**Affiliations:** Liver Unit, Department of Gastroenterology, Pomeranian Medical University in Szczecin, Unii Lubelskiej Str. 1, 71-252 Szczecin, Poland; michal.wasilewicz@pum.edu.pl

Hepatocellular carcinoma (HCC) accounts for 7% of all malignancies and about 90% of all primary liver malignancies, making it the most common type of malignant liver neoplasm [1]. HCC has an extremely poor prognosis. The mortality rate of patients with HCC is high, regardless of geographical region, and the 5 year overall survival (OS) rate of patients with this disease does not exceed 20% [1,2]. This is mainly because most HCC patients are diagnosed at a time of significant clinical advancement. This is related to the oligosymptomatic course of the disease and several common risk factors, leading to liver cirrhosis and hepatotropic viral infections. HCC is also especially prone to relapse after surgical treatment, including liver transplantation, due to its outstanding affinity for vascular expansion [1,2]. Early HCC is usually detected incidentally in different imaging procedures performed for reasons other than a suspected focal lesion in the liver (e.g., abnormal laboratory liver test results) or during surveillance programs for cirrhotic patients. Unfortunately, HCC remains a global challenge in modern oncology, with an ever-increasing incidence worldwide over the last few decades. Current annual statistics show that over 1 million new cases of HCC are diagnosed worldwide, and nearly 1 million deaths due to HCC are recorded per year [1,2,3,4]. HCC incidence increases with age, peaking at the age of 70. In most countries, the prevalence of HCC is 2–4 times higher in men than in women [1,5].

HCC is strongly associated with the ongoing process of chronic inflammation and fibrosis in the liver parenchyma. This is linked to ongoing chronic damage to the hepatocytes, and during the regeneration process, an error may arise, initiating the formation of cancer cells. Therefore, the common risk factors for HCC include predominantly liver cirrhosis, regardless of its etiology (overall, one-third of cirrhotic patients will develop HCC during their lifetime); all clinical situations with chronic inflammation and advanced fibrosis, such as chronic viral hepatitis, alcohol abuse, metabolic-associated fatty liver disease (MAFLD), hereditary metabolic disorders (e.g., hereditary hemochromatosis (HH)); and exposure to certain biological or chemical agents (e.g., nitrosamines, aflatoxins, or carbon tetrachloride) [1,5]. In recent years, numerous studies have been conducted on the genetic background of HCC, but today, almost every case of this disease appears to be spontaneous (except for rare familial aggregations of HCC) [1,2,6,7].

Hepatocellular carcinoma (HCC) is a heterogeneous malignancy originating from hepatocytes and their multipotential progenitor cells, with a very diverse nature in both its macroscopic and microscopic characteristics [8,9]. Macroscopically, we can distinguish the four basic types of HCC tumor growth as follows:

- Solitary tumor;
- Large dominant tumor along with small satellite lesions located in the immediate vicinity (no more than 2 cm) of the primary lesion, which develops due to the local spread of the tumor through the smallest branches of the portal vein;
- “Cirrhomimetic” tumor—HCC imitates many regenerative nodules in cirrhosis, characterized by numerous (from dozen to hundreds) nodules of almost the same size;
- Multiple independent HCC tumors of different sizes, without pre-dominant lesions.

The current microscopic classification of HCC is even more complicated, with nine different histological types of the disease, of which the steatotic subtype is the most common. All subtypes, except fibrolamellar carcinoma (FL-HCC), may occur in cirrhotic or non-cirrhotic livers. The FL-HCC subtype is found only in non-cirrhotic livers, and unlike all other HCC subtypes, the fibrolamellar variant has its own genetic marker defined by DNAJB1–PRKACA translocation [8]. Macrotrabecular and neutrophil-rich cancers have a worse prognosis, whereas clear-cell and lymphocyte-rich cancers have a better prognosis [8,9].

The gold standard for diagnosing HCC is contrast-enhanced radiological imaging, particularly multiphasic computed tomography (CT) or magnetic resonance imaging (MRI) of the abdomen (Gd-EOB-DTPA-enhanced), which simultaneously allows for an assessment of the extent of the disease and the possibility of resecting the HCC lesions. This is related to the characteristic pattern of post-contrast enhancement of HCC lesions in cirrhotic patients, with a strong enhancement in the arterial phase and a “wash-out” effect in the venous/delayed phase [1,10]. A complementary procedure to radiological imaging for the final diagnosis of HCC in patients without typical contrast patterns in CT/MRI examinations or in non-cirrhotic patients is a core-needle biopsy of the liver tumor [1,5]. This provides us the opportunity to state a definite diagnosis with a precise histopathological and pathogenetic assessment in terms of, for example, the availability of potential new treatment regimens using targeted immunochemotherapy. While the alpha-fetoprotein (AFP) marker may hold some diagnostic importance, its limited specificity and sensitivity in HCC, not exceeding 50–60%, prevents its use as a primary diagnostic tool [1]. Nevertheless, it can help assess the risk of poor prognosis and disease recurrence after surgical treatment [1,11].

For over the last 20 years, most HCC centers have used the BCLC (Barcelona Clinic Liver Cancer; http://www.bclc.cat (accessed on 31 July 2023)) staging system to assess the stage of HCC with the simultaneous allocation of the most appropriate therapeutic possibilities and potential outcome predictions for the patients [1,12,13]. This is crucial in taking care of HCC patients according to the decisions of the interdisciplinary team of specialists (hepatologists, oncologists, surgeons, radiologists, pathologists, palliative care specialists, etc.) in the dedicated HCC centers [1,13]. Surgical treatment—R0 resection with a margin of normal tissue (considering the area of portal vein vasculature next to the HCC lesion) or liver transplantation within the Milan criteria—has remained the only possible radical treatment for early stage HCC for many years [1,11]. Patients who are optimal candidates for this potentially radical surgery have a 5 year OS rate of 60–80%. However, only about 20–30% of patients can be offered this therapy due to the stage of the disease at the time of diagnosis [1,12]. Locoregional therapies, such as transarterial chemoembolization (TACE), selective internal radiation therapy (SIRT), or radiofrequency/microwave ablation (RFA/MVA), are therapeutic options in the palliative management of patients with advanced HCC, but the efficacy of these procedures is limited [1,7].

Over the last 15 years, along with the development of molecular and immunological research in modern clinical oncology, incredible progress has been made in HCC research and therapy. The first targeted immunochemotherapy using a multi-kinase inhibitor (MKI)—sorafenib—was introduced in HCC therapy in 2007, opening a new era in liver cancer management [14]. Cancer immunochemotherapy attacks malignant cells by activating our own immune system. The use of natural immunological mechanisms helps avoid collateral damage to normal tissues, which is one of the most serious side effects of classic chemotherapy and radiotherapy. This feature supports the rapid application of immunochemotherapy in clinical practice. The most prevalent modern immunotherapies for HCC are geared toward checkpoint inhibitors, engineered T cells, lymphocyte-promoting cytokines, and cancer vaccines. One of the best-known examples of immune checkpoint blockade (ICB) is monoclonal antibodies targeting the cytotoxic T-lymphocyte-associated protein 4 (CTLA-4) and programmed cell death 1 (PD-1) receptor [7,15,16,17]. In everyday practice, the most up-to-date treatment regimens for advanced HCC usually include the following:

- First-line therapy—atezolizumab (monoclonal antibody against PD-1 receptor) + bevacizumab (monoclonal antibody against VEGF (Vascular Endothelial Growth Factor) molecule)

OR

durvalumab (monoclonal antibody against PD-1 receptor) + tremelimumab (monoclonal antibody against CTLA-4)

OR

sorafenib (MKI) or lenvatinib (MKI)—optional for patients with contraindications to atezolizumab + bevacizumab and durvalumab + tremelimumab [13,15]

- Second-line therapy—regorafenib (MKI), cabozantinib (MKI), or ramucirumab (monoclonal antibody against VEGF receptor-2) [13,15]

In the latest research on immune-targeted drugs, sequential immunotherapies are also being investigated, which are used to prepare patients for liver transplantation by downstaging HCC lesions before listing at the liver transplant center [15,16,17,18]. However, many patients still show resistance to ICB drugs and other targeted immunotherapeutic agents [7,12]. This is exactly why we have 942 active trials (registered at www.clinicaltrials.gov—as of 31 July 2023) with different agents against ongoing liver cancer to achieve the best modern weapons (even “genetic vaccines”) for the war with HCC—one of our most insidious enemies in contemporary clinical oncology [2,7,19].
